# The Polypyrrole/Multiwalled Carbon Nanotube Modified Au Microelectrode for Sensitive Electrochemical Detection of Trace Levels of Pb^2+^

**DOI:** 10.3390/mi8030086

**Published:** 2017-03-11

**Authors:** Xuxing Zhu, Jianhua Tong, Chao Bian, Chengyao Gao, Shanhong Xia

**Affiliations:** 1 State Key Laboratory of Transducer Technology, Institute of Electronics, Chinese Academy of Sciences, Beijing 100190, China; xxze@foxmail.com (X.Z.); cbian@mail.ie.ac.cn (C.B.); gaochengyao.ok@gmail.com (C.G.); shxia@mail.ie.ac.cn (S.X.); 2 School of Electronic, Electrical and Communication Engineering, University of Chinese Academy of Sciences, Beijing 100080, China

**Keywords:** polypyrrole/multiwalled carbon nanotube, Au microelectrode, differential pulse stripping voltammetry, Pb^2+^

## Abstract

The sensitive detection of trace levels of heavy metal ions such as Pb^2+^ is of significant importance due to the health hazard they pose. In this paper, we present a polypyrrole (PPy)/multiwalled carbon nanotube (MWCNT)-modified Au microelectrode. The PPy/MWCNT composite film was electrochemically deposited on the microelectrode by cyclic voltammetry (CV). The composite film was investigated by scanning electron microscope (SEM), CV, and electrochemical impedance spectroscopy (EIS), and the results show that this film presents a uniformly distributed and web-like entangled structure and good conductivity. Differential pulse stripping voltammetry (DPSV) was applied to determine trace levels of Pb^2+^. Experimental conditions including accumulation time and deposition potential were optimized. In optimal conditions, the PPy/MWCNT-modified microelectrode performed sensitive detection of Pb^2+^ within a concentration range from 1 to 100 μg·L^−1^, and the limit of detection was 0.65 μg·L^−1^ at the signal-to-noise ratio of three.

## 1. Introduction

Currently, toxic heavy metals are a growing threat to environmental chemistry and public health. There are numerous health problems caused by the exposure of humans to heavy metal ions (Pb^2+^, Cd^2+^, Hg^2+^, etc.). Moreover, these heavy ions tend to accumulate in the body with a slow removal rate; among them, Pb^2+^—compounds of which have high toxicity—can be present for more than 20 years [1]. According to the Environmental Protection Agency (EPA), about 20% of human exposure to Pb^2+^ is through contaminated drinking water [1]. So, the trace detection of heavy metal ions such as Pb^2+^ in water is essential for human health and environmental safety [2]. High sensitivity, rapid response, and ease-of-use are keys to effective detection and are helpful to environmental protection.

Among the various detection methods, electrochemical sensors have attractive features in monitoring process because of their high sensitivity, easy operational procedures, and portability. Differential pulse stripping voltammetry (DPSV) has been seen as a good detection technology for trace-level heavy metals because it involves unique accumulation/preconcentration of analyte species contained in the solutions [3,4]. Based on the unique advantages of this method, the performance of chemically-modified electrodes could be greatly enhanced by surface modification.

Various kinds of nanomaterials have been modified on the surface of electrodes to increase sensitivity and efficiency of the determination of heavy metal ions. Multiwalled carbon nanotubes (MWCNTs) have the advantages of small size, large surface area, good conductivity, high sensitivity, and fast electron transport when used as electrode modifiers in electrochemical reactions; thus, MWCNTs have been commonly used in the fabrication and modification of various electrochemical sensors and biosensors in the past few years [5,6,7,8]. MWCNTs also have the physical features of hollow and layered structures and high aspect ratio, giving them strong abilities to adsorb heavy metal ions [9,10]. Based on this, electrochemical sensors modified by MWCNTs provide promising approaches to increasing the electrochemical response to heavy metal ions.

The modification of MWCNTs on the electrode uses various methods. The traditional methods such as physical coating method and self-assembled method result in a non-uniform distribution on the surface of the electrode and a poor sensor performance [11,12]. Composite electrochemical deposition can achieve higher uniformity and stability. The single components of the performance through synergy can also be retained [13]. Polypyrrole (PPy)—one of the most important conducting polymers—has high electrical conductivity, good electrochemical activity, and long-term environmental stability, meaning that PPy is very suitable for composite films [14,15]. 

In this work, we present a polypyrrole (PPy)/MWCNT-modified Au microelectrode for the electrochemical detection of Pb^2+^. The PPy/MWCNT composite films were modified on a Au microelectrode via electrochemical deposition. Due to the large surface area and porous structure, PPy acts not only as the backbone of the nanomaterial, it also performs a great absorption property for heavy metal ions [16]. We use the PPy/MWCNT modified microelectrode, achieving the electrochemical detection of Pb^2+^. The modification process was investigated, and the PPy/MWCNT-modified microelectrode was fabricated. The electrochemical characteristics were examined, and the experimental conditions of DPSV were optimized for the detection of Pb^2+^.

## 2. Experimental

### 2.1. Reagents and Apparatus

Pyrrole was purchased from Aladdin (Shanghai, China). Multiwalled carbon nanotubes were purchased from Nanjing XFNANO Materials Tech Co., Ltd. (Nanjing, China). Lead standard stock solution (100 mg/L Pb^2+^ in 3% nitric acid) was purchased from the China National Research Centre for Certified Reference Material. Lead samples were diluted from the stock solution with different concentrations. All other chemicals were of analytical grade and were used without further purification. Doubly deionized water was used throughout the experiments.

All electrochemical tests were conducted by Gamry Reference 600 electrochemical workstation (Gamry Instruments Co., Ltd., Warminster, PA, USA) using a three electrode system based on a self-fabricated modified Au microelectrode as working electrode, an on-chip Au electrode as counter electrode, and a commercial saturated Ag/AgCl as reference electrode. Field-emission scanning electron microscope (FE-SEM; S-4800) produced by Hitachi (Tokyo, Japan) was used to check the morphologies.

### 2.2. Microelectrode Fabrication

**Figure 1:**
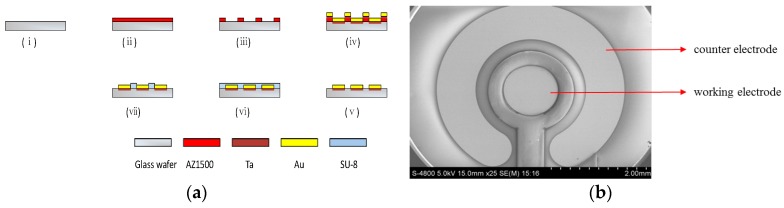
The (**a**) fabrication process: (i) glass wafer cleaning; (ii) AZ1500 patterning; (iii) photolithography; (iv) Ta/Au layer sputtering; (v) lift off process; (vi) SU-8 patterning; (vii) photolithography; and (**b**) scanning electron microscope (SEM) image of the microelectrode chip.

The microelectrode chips were fabricated by microelectromechanical systems (MEMS) technique, and the fabrication process is shown in Figure 1. An Au disk electrode with area of 1 mm^2^ and an Au counter electrode were fabricated on glass substrate by standard photolithographic, sputtering, and lift-off process. The thickness of Au and Ta (adhesive layer) were 2000 and 200 Å, respectively. The negative photoresist (SU-8) was used to form the insulting layer. After dicing the patterned glass into individual chips with a size of 8 mm × 8 mm, the microelectrodes were wire-bonded and encapsulated on printed circuit board strips. Figure 1b illustrates an SEM image of the fabricated microelectrode.

### 2.3. Preparation of PPy/MWCNT-Modified Microelectrode

MWCNTs aggregate easily and are difficult to disperse into solutions as a result of substantial Van der Waals attractions between them [17]. So, MWCNTs need to be dispersed in sodium dodecyl sulfate (SDS) before modification process. Aqueous MWCNTs dispersion was prepared by adding MWCNTs into SDS dispersion and sonication procedure, in which the mass percent of MWCNT was 10%. Then, the composite solution was prepared containing 10 g·L^−1^ MWCNTs, 0.1 M pyrrole, and 0.1 M NaCl.

Before modification, the Au microelectrode was cleaned in 0.01 M H_2_SO_4_ by cyclic voltammetry (CV) within 0–1.5 V until the curve was stable. The PPy/MWCNT composite film was electrochemically deposited in the presence of NaCl (acting as supporting electrolyte). The deposition process was performed by CV within the oxidation correction interval of pyrrole (−0.6–0.8 V). The deposition cycles used at the end of the experiment were 15 cycles.

### 2.4. Electrochemical Measurements

Trace levels of Pb^2+^ were detected by DPSV under the optimized conditions. All solutions were stored in a 20 mL Teflon bottle. The detection was conducted in 0.1 M HCl under the potential range of −0.45 to 0.05 V. DPSV parameters were optimized such that Pb^2+^ was deposited for 300 s (accumulation time) at −0.9 V (deposition potential). Prior to the next determination, the modified microelectrode was cleaned for 300 s at 0.4 V in 0.1 M HCl. Repeated operation was conducted until Pb^2+^ was cleaned out completely.

## 3. Results and Discussion

### 3.1. Morphologies of Modified Microelectrode

**Figure 2:**
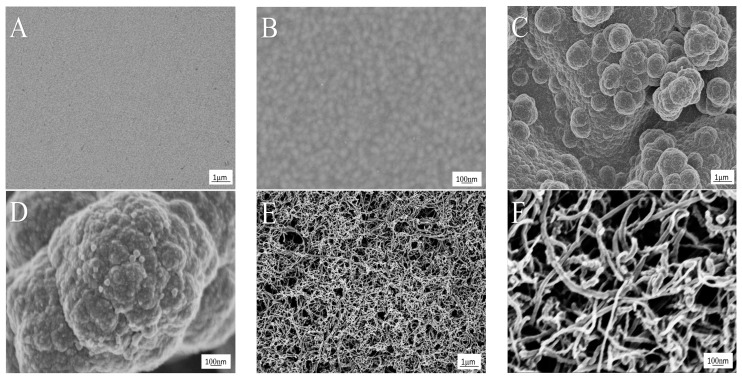
SEM images of (**A**,**B**) bare Au; (**C**,**D**) polypyrrole (PPy)/Au; (**E**,**F**) PPy/MWCNT/Au (MWCNTs: multiwalled carbon nanotubes). The magnifications are (A,C,E) 10 k; and (B,D,F) 60 k.

SEM was used to investigate the surface morphology and structure of the modified microelectrodes, as shown in Figure 2. Figure 2A,B shows the micrograph of a bare Au microelectrode treated by clean procedures, in which the flat uniform sputtered gold particles can be seen. Figure 2C,D shows the micrograph of PPy/Au. The PPy film was deposited by CV in 0.1 M NaCl within the interval from −0.6 to 0.8 V for 15 cycles. As can be observed, the PPy film displayed rough surface, porous and three-dimensional structure, indicating that PPy was electrodeposited on the surface of the Au microelectrode. As reported, when a small anion such as Cl^−^ acts as counterion, the PPy film surface presents rougher and more obviously porous [18]. Based on this, more MWCNTs can adequately distribute on top of the surface. Figure 2E,F shows the micrograph of PPy/MWCNT/Au, in which multi-layer of MWCNTs distribute on the surface extensively and uniformly. The composite film presents a three-dimensional and web-like entangled structure.

### 3.2. Characterization of Modified Microelectrode

**Figure 3:**
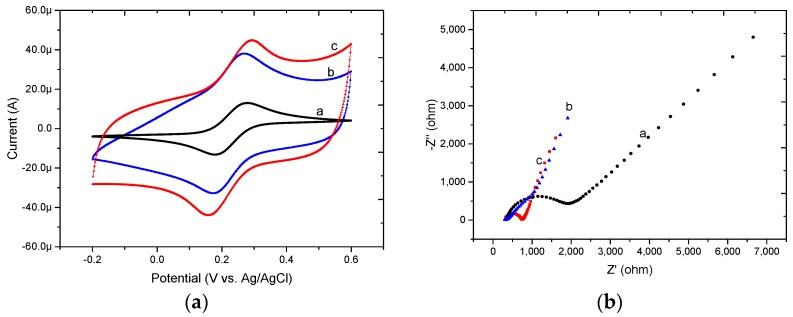
(**a**) Cyclic voltammetry (CV); and (**b**) electrochemical impedance spectroscopy (EIS) spectrums of bare Au (curve a), PPy/Au (curve b), and PPy/MWCNT/Au (curve c) microelectrodes in 5 mM [Fe(CN)_6_]^3^^−^/[Fe(CN)_6_]^4^^−^ containing 0.1 M KCl.

As shown in Figure 3a, CV was conducted to represent the electrochemical characterization of the microelectrode after modification. The bare Au microelectrode (curve a) shows a reversible redox behavior, which indicates that there is a flexible electron transfer between [Fe(CN)_6_]^3−^ and the microelectrode. After the fabrication of the PPy film and the PPy/MWCNT composite film on the microelectrode, PPy has a high surface/volume ratio leading to the increased current contrasted to the bare Au. MWCNTs have excellent adsorption abilities to increase the effective area of microelectrode surface in response to [Fe(CN)_6_]^3−^, resulting in further increased current in the case of PPy/MWCNT/Au (curve c) compared to PPy/Au (curve b). 

Besides, the results of impedance on the Au microelectrode with different layers were shown in Figure 3b, conducted by electrochemical impedance spectroscopy (EIS). The electron transfer resistance (Ret) is represented by the semicircle diameter at higher frequencies, and the diffusion process corresponds to the linear part at lower frequencies [19]. It can be seen that the bare Au microelectrode exhibits a good characteristic of a diffusion-limited electrochemical process (curve a). The Ret value decreased obviously, as observed in the case of PPy/Au (curve b), suggesting faster electron transfer kinetics of [Fe(CN)_6_]^3−/4−^ on the microelectrode surface. It can be seen that the Ret value of PPy/MWCNT/Au (curve c) was between bare Au and PPy/Au, indicating the presence of MWCNTs. 

### 3.3. Electrochemical Behaviors of Pb^2+^ at Various Microelectrodes

**Figure 4:**
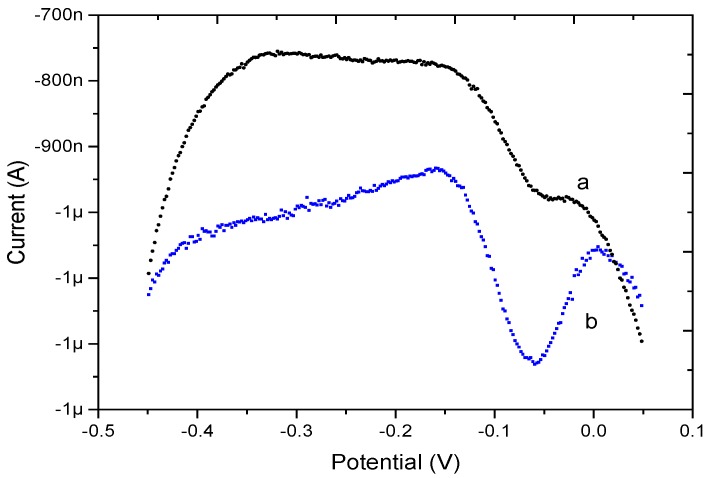
Differential pulse stripping voltammetry (DPSV) responses of bare Au (curve a) and PPy/MWCNT/Au (curve b) in the presence of 10 μg·L^−1^ Pb^2+^.

The DPSV electrochemical responses of bare Au microelectrode and PPy/MWCNT-modified Au microelectrode to 10 μg·L^−1^ Pb^2+^ were investigated for comparison, as shown in Figure 4. The peak current response of the bare Au (curve a) was about 180 nA. By contrast, the peak current response of PPy/MWCNT/Au (curve b) was about 1.2 μA. Due to the good Pb^2+^ enrichment effect of MWCNTs, the PPy/MWCNT/Au can increase the response to Pb^2+^ at low concentrations of Pb^2+^. Compared with the bare Au microelectrode, the PPy/MWCNT-modified microelectrode exhibited a higher current response at low concentration.

### 3.4. Optimization of Experimental Conditions

DPSV was used to detect trace levels of Pb^2+^ because of its high sensitivity and linearity [20]. The electrochemical analysis included three steps: (i) accumulation process; (ii) electrochemical reductive process; (iii) electrochemical oxidative process [21]. For the purpose of achieving high sensitivity for trace Pb^2+^ detection with PPy/MWCNT/Au, the experimental conditions including accumulation time and deposition potential were optimized. 

#### 3.4.1. Optimization of Accumulation Time

**Figure 5:**
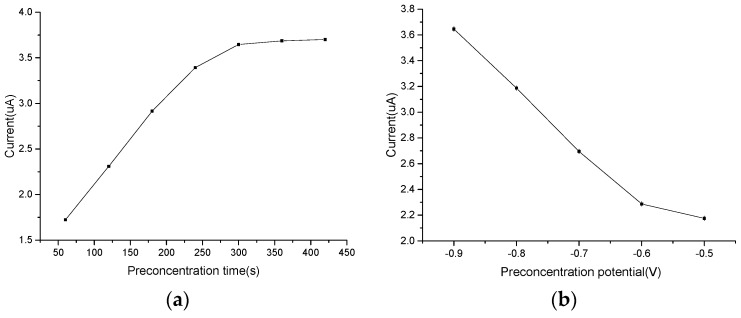
DPSV carried out on the PPy/MWCNT/Au in the presence of 100 μg·L^−1^ Pb^2+^ in 0.1 M HCl of (**a**) different accumulation time (deposition potential: −0.9 V); and (**b**) different deposition potential (accumulation time: 300 s).

The accumulation time affects the amount of absorbed Pb^2+^ on the surface of modified electrode, and then affects the detection sensitivity. To improve the detection sensitivity, we investigated the influence of accumulation time at −0.9 V. As shown in Figure 5a, the accumulation time was studied from 60 to 420 s. Before 300 s, Peak currents of Pb^2+^ increased sharply with increasing accumulation time. After 300 s, with the time further increasing, the peak current increased slowly, indicating that 300 s is sufficient for the detection by the PPy/MWCNT/Au microelectrode. Thus, we selected 300 s as the optimal accumulation time for Pb^2+^ detection.

#### 3.4.2. Optimization of Deposition Potential

The deposition potential is vital to achieving a high microelectrode sensitivity. Thus, the effect of deposition potential was investigated by applying varying potentials. As can be observed in Figure 5b, the peak currents increased with deposition potential varying from −0.5 to −0.9 V and reached a maximum at potential −0.9 V. During the experiment, when deposition potential reached −1.0 V, apparent hydrogen evolution occurred on the surface of the microelectrode, and the peak current decreased. In addition, the modified films were damaged due to the hydrogen evolution, and it was difficult to repeat the detection. With the deposition decreased further, hydrogen evolution was increasingly obvious. So, −0.9 V was chosen as the optimal accumulation potential for the Pb^2+^ detection.

### 3.5. Electrochemical Determination of Pb^2+^

**Figure 6:**
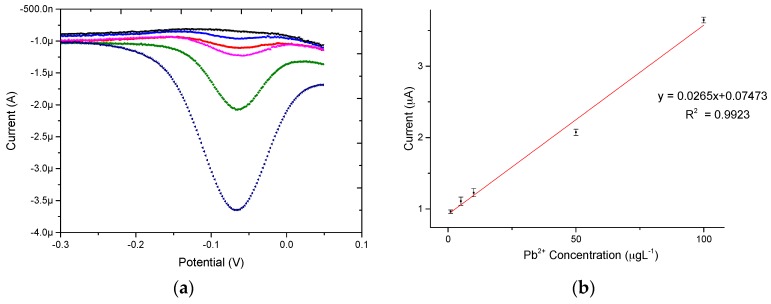
(**a**) DPSV responses of the PPy/MWCNT/Au for the detection of different concentrations of Pb^2+^ (from 1 to 100 μg·L^−1^) in 0.1 M HCl; and (**b**) the corresponding linear calibration plots of stripping peak currents for Pb^2+^.

Under the optimized conditions described above, the electrochemical detection of Pb^2+^ at different concentrations using the PPy/MWCNT/Au microelectrode were performed by DPSV. The determination results of Pb^2+^ by DPSV with different concentrations from 1 to 100 μg·L^−1^ are shown in Figure 6. It can be observed that the well-defined peak currents increased linearly with the increased concentration of Pb^2+^, and the corresponding linear regression equation is represented as *y* = 0.0265*x* + 0.07473, with the correlation coefficient of 0.9923. The detection limit for Pb^2+^ was calculated to be 0.65 μg·L^−1^ (signal-to-noise ratio (*S*/*N*) = 3), which means the PPy/MWCNT/Au method exhibited a wide concentration range with a low detection limit for the detection of Pb^2+^. The PPy/MWCNT-modified microelectrode performed a high sensitivity for the detection of Pb^2+^ within the concentration range from 1 to 100 μg·L^−1^.

As shown in Table 1, good linearity was acquired within the 1–100 μg·L^−1^ range. The limit of detection (LOD) was 0.65 μg·L^−1^ at the signal-to-noise ratio of three, which is comparable to other electrochemical detection methods. This indicates that the PPy/MWCNT-modified Au microelectrode is an effective method for highly sensitively detecting trace levels of Pb^2+^.

### 3.6. Interference Study

Interferences similar to Pb^2+^ were investigated to evaluate the specificity of PPy/MWCNT/Au to Pb^2+^. We used 100 μg·L^−1^ Pb^2+^ and 50-fold concentration interference metal ions for detection, including Ca^2+^, Mg^2+^, Mn^2+^, Ni^2+^, Co^2+^. As shown in Table 2, the similar metal ions did not interfere with the specificity of the Pb^2+^ determination. The results indicated good specificity of Pb^2+^ with the existence of interferences, and confirmed the practicability in application. 

## 4. Conclusions 

In this paper, a composite electrochemical deposition method for preparing a PPy/MWCNT nanocomposite-modified electrode was proposed. The PPy/MWCNT composite was successfully modified on the Au microelectrode fabricated by the MEMS technique. The film presented a uniform web-like entangled structure and possessed a better stability and higher sensitivity. Compared with a bare Au microelectrode, the composite film-coated Au microelectrode greatly improved the sensitivity of determining Pb^2+^ at low concentrations. The PPy/MWCNT/Au microelectrode conducted sensitive detection of Pb^2+^ within the concentration range from 1 to 100 μg·L^−1^, and the limit of detection was 0.65 μg·L^−1^ (*S*/*N* = 3). 

## Figures and Tables

**Table 1:** Electrode performance for measuring Pb^2+^.

Electrode	Linearity Range (μg·L^−1^)	Correlation Coefficient	LOD (μg·L^−1^)	Reference
MWCNTs/GCE	4.14–200	0.99	0.83	[22]
Crown/GCE	5.0–186.5	0.99	1.5	[23]
b^2^SPE	5–110	0.99	1.10	[24]
PPy/MWCNT/Au	1–100	0.9923	0.65	This work

GCE: glass carbon electrode; b^2^SPE: back-to-back screen-printed electrode; LOD: limit of detection; PPy: polypyrrole; MWCNT: multiwalled carbon nanotube.

**Table 2:** The effect of various interferences on the peak current of Pb^2+^.

Interferences	Signal Change (%)
Ca^2+^	+4.5
Mg^2+^	+3.5
Mn^2+^	+2.6
Ni^2+^	−3.1
Co^2+^	−2.8
